# Relevance of well-being, resilience, and health-related quality of life to mental health profiles of European adolescents: results from a cross-sectional analysis of the school-based multinational UPRIGHT project

**DOI:** 10.1007/s00127-021-02156-z

**Published:** 2021-08-21

**Authors:** Carlota Las-Hayas, Maider Mateo-Abad, Itziar Vergara, Irantzu Izco-Basurko, Ana González-Pinto, Silvia Gabrielli, Iwona Mazur, Odin Hjemdal, Dora Gudrun Gudmundsdottir, Hans Henrik Knoop, Anna Sigríður Olafsdottir, Ane Fullaondo, Nerea González, Javier Mar-Medina, Dominik Krzyżanowski, Roxanna Morote, Frederick Anyan, Mette Marie Ledertoug, Louise Tidmand, Unnur Björk Arnfjord, Ingibjorg Kaldalons, Bryndis Jona Jonsdottir, Esteban de Manuel Keenoy, the UPRIGHT Consortium, Iñaki Zorrilla-Martínez, Patricia Pérez-Martínez-de-Arrieta, Igor Larrañaga, Sara Carbone, Silvia Rizzi, Valeria Donisi, Hrefna Pálsdóttir, Alda Ingibergsdóttir

**Affiliations:** 1 Kronikgune Institute for Health Services Research, Ronda de Azkue 1 torre del Bilbao Exhibition Centre, 48902 Barakaldo, Basque Country Spain; 2 REDISSEC (Health Services Research On Chronic Patients Network), Barakaldo, Basque Country Spain; 3 Biodonostia Health Research Institute, San Sebastián, Spain; 4 Osakidetza Basque Health Service, Araba University Hospital, Vitoria - Gasteiz, Basque Country Spain; 5 University of the Basque Country UPV/EHU, Leioa, Spain; 6 CIBERSAM (CIBER of Mental Health Area), Madrid, Spain; 7 Bioaraba Health Research Institute, Vitoria-Gasteiz, Spain; 8 Bruno Kessler Foundation, Via Santa Croce 77, 38122 Trento, Italy; 9 Daily Centre for Psychiatry and Speech Disorders, Wrocław, Poland; 10 Wroclaw Medical University, Wrocław, Poland; 11 Norwegian University of Science and Technology, Hogskoleringen 1, 7491 Trondheim, Norway; 12 Directorate of Health, Baronsstig 47, 101 Reykjavik, Iceland; 13 Aarhus University, Nordre Ringgade 1, C 8000 Aarhus, Denmark; 14 Optentia Research Focus Area, North-West University, P O Box 1174, Vanderbijlpark, 1900 South Africa; 15 School of Education, University of Iceland, Saemundargotu 2, 101 Reykjavik, Iceland; 16 Osakidetza Basque Health Service, Barrualde-Galdakao Integrated Health Organisation, Galdakao, Spain; 17 Research Unit, Osakidetza Basque Health Service, Debagoiena Integrated Health Organisation, Arrasate-Mondragón, Spain; 18 Division of Medical Social Science, Wroclaw Medical University, Wrocław, Poland; 19 Department of Health, Lower Silesia Voivodeship Marshal Office, Wrocław, Poland; 20 Catholic University of Peru, Avenida Universitaria s/n, 18, Lima, Peru

**Keywords:** Youth, Well-being, Mental disorders, Mental health promotion, Prevention, School interventions

## Abstract

**Purpose:**

The existing evidence suggests that a complete evaluation of mental health should incorporate both psychopathology and mental well-being indicators. However, few studies categorize European adolescents into subgroups based on such complete mental health data. This study used the data on mental well-being and symptoms of mental and behavioral disorders to explore the mental health profiles of adolescents in Europe.

**Methods:**

Data collected from adolescents (*N* = 3767; mean age 12.4 [SD = 0.9]) from five European countries supplied the information on their mental well-being (personal resilience, school resilience, quality of life, and mental well-being) and mental and behavioral disorder symptoms (anxiety, depression, stress, bullying, cyber-bullying, and use of tobacco, alcohol, or cannabis). Multiple correspondence analysis and cluster analysis were combined to classify the youths into mental health profiles**.**

**Results:**

Adolescents were categorized into three mental health profiles. The "poor mental health" profile (6%) was characterized by low levels of well-being and moderate symptoms of mental disorders. The "good mental health" profile group (26%) showed high well-being and few symptoms of mental disorders, and the "intermediate mental health" profile (68%) was characterized by average well-being and mild-to-moderate symptoms of mental disorders. Groups with higher levels of well-being and fewer symptoms of mental disorders showed lower rates of behavioral problems. Mental well-being indicators strongly contributed to this classification.

**Conclusion:**

Adolescents with the "intermediate" or "poor" mental health profiles may benefit from interventions to improve mental health. Implications for school-based interventions are discussed.

**Trial registration number (TRN) and date of registration:**

ClinicalTrials.gov Identifier: NCT03951376. Registered 15 May 2019.

**Supplementary Information:**

The online version contains supplementary material available at 10.1007/s00127-021-02156-z.

## Introduction

Adolescence is the time when many of the skills that contribute to well-being (e.g., autonomy, self-control, social interaction, and learning) are developed, and the foundations of the lifelong mental health are laid down [1]. Half of the mental disorders diagnosed in adulthood originate during this period [1].

Traditionally, mental health was an inferred by-product of the absence of mental disorder [2]. Currently, mental health has been defined as “a state of well-being in which the individuals realize their abilities, can cope with the normal stresses of life, work productively and fruitfully, and contribute to their community” [3]. Some studies have linked subjective well-being with several positive health and social outcomes [4]; others have demonstrated that positive mental health and mental disorders are not mutually exclusive. Instead, these two conditions are generally inversely associated [5], differ in scope [5, 6], and have different drivers [7]. Thus, to obtain a complete assessment of the mental health status of adolescents, it is necessary to evaluate both the negative aspects (related to their vulnerability to mental disorders) and the positive factors (related to their well-being).

Tudor [8] and, later, Keyes [5, 9] have presented the dual continua model of well-being and mental disorders. Its validity has been supported by a considerable body of empirical research [10]. The model shows that subjective well-being and mental disorder symptoms are separable conditions contributing to child function predictions. It describes four mental health profiles obtained by combining these two dimensions. Several studies have explored mental health profiles integrating the mental disorder-related and positive mental health factors [11–13]. However, a few studies categorize European adolescents into subgroups based on such complete mental health data. The main aim of the current study, which is part of a wider research project, UPRIGHT (Universal Preventive Resilience Intervention Globally implemented in schools to improve and promote mental Health for Teenagers), is to obtain the mental health profiles for a large sample of European adolescents for the years 2018–2019. The study explores the positive factors (well-being, resilience, and health-related quality of life) and, mental disorder-related factors (symptoms of anxiety, depression, stress, behavioral problems, and substance abuse), and the associations between them.

## Method

The aim of the UPRIGHT project is to develop an effective, universal, and holistic school-based intervention. The program is designed to be applied in the school years corresponding to the ages between 12 and 14, regardless of risk condition, and includes their families and the school communities. It is currently being implemented, and its effectiveness tested in different regions from 5 European countries: Basque Country (Spain), Lower Silesia (Poland), Trentino (Italy), Reykjavik area (Iceland), and the regions of North-Sealand, West-Sealand, Funen, North Jutland, and Eastern Jutland (Denmark). The UPRIGHT project is funded by the European Union's Horizon 2020 research and innovation program under grant agreement Nº 754919. The research protocol has been published elsewhere [14].

Here, we present the baseline data collected from all adolescents taking part in the UPRIGHT project before implementing the UPRIGHT intervention. Thirty-four schools from five countries have participated, representing a mixture of urban, rural, socioeconomically disadvantaged, and non-disadvantaged areas.

### Data collection

The self-reported information was collected from September 2018 to the end of 2019. All questionnaires had been already validated in all regional languages and were distributed in the classrooms during school hours. They could be completed either on paper or online (Qualtrics, Provo, UT), and a member of the school staff was always present during the completion of the questionnaires.

The data were pseudonymized, i.e., separated from its direct identifiers, so linking the results to a person was only possible using additional information. This information was kept secure and separate from processed data to ensure non-attribution [15].

### Measures

The recorded socio-demographic characteristics consisted of gender, year of birth, country of birth, number of children living in the household, and order of birth.

Positive mental health-related outcomes were assessed using the following scales

Mental well-being was measured using the 14-item Warwick-Edinburgh Mental Well-Being Scale (WEMWBS) [16]. Higher scores (score range from 14 to 70) indicate higher levels of positive mental well-being. The levels of mental well-being of students can be interpreted as high (scores > 60), average (scores around 51), and low (scores < 40) [16]. The Cronbach's alpha was 0.85 95% CI [0.84, 0.85] for the current sample.

The health-related quality of life (HRQoL) was assessed employing the 10-item KIDSCREEN scale [17], which is used for children and adolescents aged 8–18. The items assess satisfaction with family life, peers, and school life. The KIDSCREEN-10 instrument provides a singular index for global HRQoL, in the range from 0 to 100. The normative score for the scale is 71.9 [18], and the higher the score, the higher the HRQoL. The Cronbach's alpha reached 0.82, 95% CI [0.82, 0.83], for the current sample.

To measure the principal protective factors of resilience, a 28-item resilience scale for adolescents, READ [19], was used. The READ measures five factors: personal competence (PC), social competence (SC), structured style (SS), social resources (SR), and family cohesion (FC). The total score and each factor score range from 1 to 5. Higher scores reflect higher resilience. Cronbach's alpha of 0.93, 95% CI [0.93, 0.93], was obtained for the full scale.

The 5-item School Resilience Scale [20] measures five interrelated aspects of the school community, considered precursors of resilience and mental well-being of young people: (1) positive relationships, (2) belonging, (3) inclusion, (4) participation, and (5) mental health awareness of all the members of the school community. The total score ranges from 1 to 5, with higher scores indicating higher levels of school resilience. Cronbach's alpha was 0.72, 95% CI [0.71, 0.74].

Mental disorder-related factors were assessed using the following scales

The 4-item Perceived Stress Scale (PSS-4; [21]) are designed to assess the feelings of being overwhelmed and unable to control or predict life events. Scores range from 0 to 16, with higher scores corresponding to higher levels of perceived stress. The norm value for interpreting the PSS-4 scores was 5.43 [22]. Cronbach's alpha reached 0.61, 95% CI [0.59, 0.63].

The 9-item version of the Patient Health Questionnaire (PHQ-9) [23, 24] gives a measure of depression; it also helps assess the severity of depressive disorders. The total score ranges from 0 to 27. The severity of the disorder can be interpreted as (0–4) minimal, (5–9) mild, (10–14) moderate, (15–19) moderately severe, or (20–27) severe. Cronbach’s alpha was 0.79, 95% CI [0.78, 0.8].

The 7-item Generalized Anxiety Disorder Scale (GAD-7) [25, 26] is a screening tool for detecting generalized anxiety disorder. The total score ranges from 0 to 21. The severity of the disorder can be interpreted as minimal (0–4), mild (5–9), moderate (10–14), or severe (15–21). Cronbach’s alpha was 0.84, 95% CI [0.83, 0.84] for the current sample.

The examined conduct problems were the violent behavior and frequency of substance use as measured employing the 8-item screen used in WHO's Health Behavior in School-Aged Children survey (HBSC) [27]. Violent behavior was assessed considering five items measuring the frequency of physical fights in the preceding 12 months, frequency of being bullied or cyber-bullied in the preceding 2 months, and taking part in a bullying or cyber-bullying episode. Substance use assessment included 4 items examining the frequency of lifetime use of tobacco, alcohol, and cannabis.

### Statistical analysis

A comparison between female, male, and non-binary genders was performed. Categorical variables were presented as frequencies and percentages (%) and continuous variables as means and standard deviations (SD). The Chi-squared test was used to compare categorical variables and ANOVA for continuous variables. Correlation between the continuous variables was assessed employing the Pearson correlation coefficient and are provided as Online Resource (Table 4). The differences were considered statistically significant at *p* < 0.050.

A combination of multiple correspondence analysis (MCA) and cluster analysis was employed to characterize the associations between all the mental health-related variables. These two multivariate techniques are widely utilized in medical research to obtain the profile associations based on the similarities of the variables of interest [28].

The MCA is a reduction method characterizing the information for various categorical variables into dimensions explaining the maximum variability levels for the variables included in the analysis [29]. The MCA was employed to identify subjacent relationships between the main variables included in the study. They were included in the analysis as categorical variables, using the categorizations of the scales (explained in the measures section). Continuous variables without any previously defined categorization were grouped into equal segments, obtaining a maximum of five categories. Then, the main dimensions of the MCA were graphically represented in a map, and each category of the variables was plotted as a point. The closer the points are, the stronger the association between the categories.

Cluster analysis was used to divide all participants into groups, based on the main dimensions provided by the MCA, i.e., the association between the variables. The classification was made employing a hierarchical cluster analysis, according to proximity criteria using Euclidean distance, and a k-means non-hierarchical cluster analysis, following the k-means algorithm. The number of clusters was chosen by selecting the best relationship between the appropriate number of clusters observed in the dendrogram and the Calinski–Harabasz index value. To assess the internal cluster quality, cluster stability in the optimal solution was analyzed using Jaccard bootstrap values (100 runs). Clusters were considered highly stable for average Jaccard similarities of 0.85 or higher [30, 31]. The obtained clusters were displayed in the geometrical space constructed using the MCA dimensions. A comparison between clusters was performed; the Chi-squared test was used to compare categorical variables and ANOVA for continuous variables.

Statistical analyses were carried out using the free statistical software R (version 3.6.1); the "ca" package was used for MCA and "stats" and "fpc" for cluster analysis.

## Results

A total of 3767 adolescents (mean age 12.4 [SD = 0.9] years) completed the questionnaires. Among them, 25% were from Italy, 23% from Iceland, 21% from Poland, 20% from Spain, and 11% from Denmark. Table 1 shows socio-demographic information and a general description of mental health-related outcomes for the adolescents and differences by gender: female (50.9%), male (48.6%), and non-binary (0.5%). Overall, the male adolescents reported the highest positive mental health, including the highest mean scores for well-being. The HRQoL and individual and school resilience results in this group also disclosed fewer mental disorder-related factors such as perceived stress, depression, and anxiety symptoms compared with the female and non-binary participants. The female subjects reported less involvement in bullying cases and substance use than the male subgroup. The adolescents who self-identified as non-binary gender reported the worst values for almost all the outcomes (Table 1). Inter-group differences between resilience scores, although statistically significant, were not relevant as these differences were close to 0.

**Table 1:** Description of the sample and differences by gender, showing socio-demographic characteristics, self-reported positive mental health outcomes, and mental disorder-related outcomes

	Total	Female	Male	Non-binary	*p *value
Sample size, *n* (%)	3767	1907 (51%)	1820 (49%)	19 (0.5%)	
Socio-demographic characteristics, *n* (%)					
Age, mean (SD)	12.4 (0.9)	12.4 (0.9)	12.3 (0.9)	13.1 (1.1)	0.001
Living with other children					0.001
No	951 (25%)	477 (25%)	464 (26%)	2 (10%)	
With 1 child	1852 (50%)	912 (48%)	926 (51%)	7 (37%)	
With 2 or more children	936 (25%)	509 (27%)	415 (23%)	10 (53%)	
Position born (oldest)	1603 (44%)	810 (43%)	777 (44%)	7 (37%)	0.942
Born in the country of residence	3443 (92%)	1747 (92%)	1666 (92%)	13 (68%)	0.004
Positive mental health outcomes, mean (SD)					
Mental well-being	50.9 (8.1)	50.4 (8)	51.5 (8)	43.6 (8.5)	< 0.001*
Health-related quality of life	69.4 (15.6)	67.2 (16)	71.9 (14.6)	53.5 (20.5)	< 0.001*
Resilience	3.8 (0.6)	3.8 (0.6)	3.8 (0.6)	3.3 (0.8)	< 0.001*
Personal competence	3.6 (0.7)	3.6 (0.7)	3.7 (0.7)	3.2 (0.9)	< 0.001*
Social competence	3.7 (0.7)	3.7 (0.7)	3.8 (0.8)	3.5 (1)	0.304
Structured style	3.4 (0.8)	3.4 (0.8)	3.5 (0.8)	3 (0.8)	0.015
Social resources	4.2 (0.7)	4.2 (0.7)	4.1 (0.7)	3.6 (0.7)	< 0.001*
Family cohesion	4 (0.8)	4 (0.8)	4 (0.7)	3.4 (1.2)	0.002
School resilience	3.7 (0.8)	3.7 (0.7)	3.6 (0.8)	2.5 (0.9)	< 0.001*
Mental disorder-related outcomes, mean (SD)					
Perceived stress	5.7 (2.9)	6 (2.9)	5.4 (2.8)	8.4 (3.4)	< 0.001*
Depression symptoms	6.5 (4.7)	7 (4.9)	5.9 (4.4)	10.9 (7)	< 0.001*
Moderate-to-severe, *n* (%)	775 (22%)	443 (25%)	318 (19%)	10 (56%)	< 0.001*
Anxiety disorder	5.5 (4.4)	6.1 (4.6)	4.8 (4.1)	9.8 (5.7)	< 0.001*
Moderate-to-severe, *n* (%)	623 (17%)	389 (21%)	222 (13%)	8 (50%)	< 0.001*
Conduct problems					
Fighting and bullying, *n* (%)					
Physical fight (last year)	1227 (33%)	361 (19%)	842 (47%)	13 (68%)	< 0.001*
Bullying (last 2 months)					
Bullied	563 (15%)	239 (13%)	314 (17%)	9 (50%)	< 0.001*
Cyber-bullied	346 (9%)	167 (9%)	169 (10%)	6 (32%)	0.006
Bullying others	401 (11%)	149 (8%)	242 (13%)	6 (32%)	< 0.001*
Cyber-bullying others	278 (7%)	117 (6%)	151 (8%)	6 (32%)	< 0.001*
Substance use (ever), *n* (%)					
Smoking tobacco	194 (5%)	69 (4%)	114 (6%)	6 (32%)	< 0.001*
Drinking alcohol	609 (16%)	239 (13%)	355 (20%)	8 (42%)	< 0.001*
Getting drunk	112 (3%)	36 (2%)	69 (4%)	5 (26%)	< 0.001*
Using cannabis	64 (2%)	19 (1%)	39 (2%)	5 (26%)	< 0.001*

Categorical data are presented as frequencies and percentages, n (%), and continuous data as means and standard deviation (SD); *p* value for differences between the three gender categories. *Statistically significant difference (p < 0.050), only considering comparisons between females and males. Percentages of missing values for all variables ranged from 1.2 to 7.6%. Scales: mental well-being (WEMWBS) [16], range 14–70; health-related quality of life (KIDSCREEN) [18], range 0–100; resilience (READ) [19], range 1–5; school resilience [20], range 1–5; perceived stress (PSS-4) [21], range 0–16; depression (PHQ-9) [23], cut-off point between moderate and severe, ≥ 10; Anxiety disorder (GAD) [26], cut-off point between moderate and severe, ≥ 10; conduct problems (subscales of HBSC) [27]

**Fig. 1:**
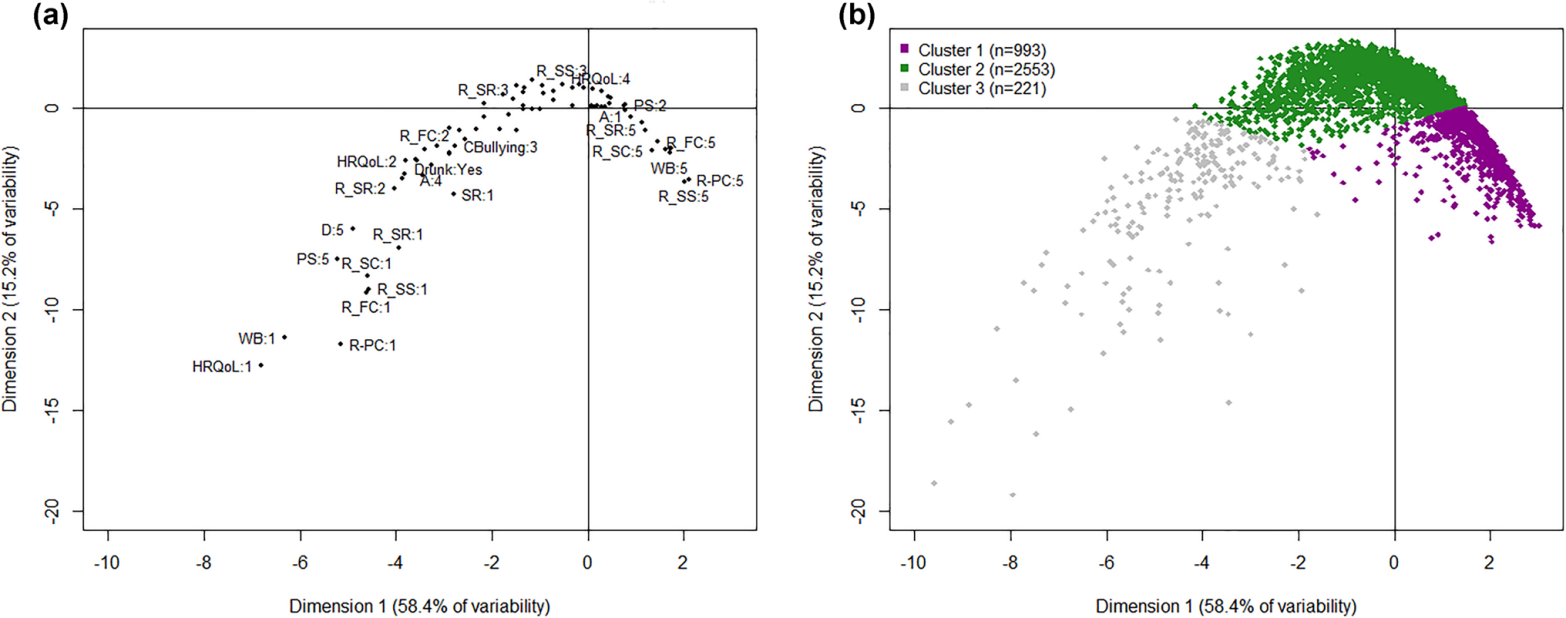
Graphical representation of the two dimensions of the multiple correspondence analysis (MCA) and the cluster analysis. **a** Black dots represent the categories of mental health variables and the conduct problems included in the MCA; only the most representative entries are labeled as "scale: category". The closer the points, the stronger the association between them. The categories are numbered from the lowest response options of the scale, 1, to the highest option (depending on the variable, from 3 to 5). Average values are around the middle of the map. The first dimension identified the gradient of mental health status, from the worst values (left) to the best values (right), and it explains 58.4% of the variability. The second dimension identifies the extreme values of the scales (down) and explains 15.2% of the variability. *WB* mental well-being, *HRQoL* health-related quality of life (KIDSCREEN-10), *R-PC* resilience-personal competence, *R-SC* resilience-social competence, *R-SS* resilience-structured style, *R-SR* resilience-social resources, *R-FC* resilience-family cohesion, *SR* school resilience, *PS* perceived stress, *D* depression, *A* anxiety disorder, *Fight* involved in a physical fight during last 12 months, *Bullying* involved bullying others during last 2 months, *CBullied* cyber-bullied during last 2 months, *CBullying* involved in cyber-bullying others during last 2 months, *Bullied* bullied during last 2 months, *Smoking* ever-smoked tobacco, *Drinking* ever drunk alcohol, *Drunk* ever become very drunk, *Cannabis* ever used cannabis. **b** Cluster 1: represents the distribution of adolescents with "good mental health"; Cluster 2: adolescents with "intermediate mental health"; and Cluster 3: participants with "poor mental health"

The underlying relationships between the different scales were also studied using the MCA analysis. The two most meaningful MCA dimensions explained, overall, 73.6% of the variability of the data. These were the first dimension, with the gradient of the mental health status, and the second dimension, identifying the extreme values of the scales (58.4% and 15.2%, respectively; see Fig. 1a). The contribution of each variable to the construction of the two dimensions is shown in Table 2. Positive mental health variables contributed more to the definition of the two MCA dimensions than mental disorder-related factors (above the expected average of 5%). In particular, four positive mental health variables (HRQoL, mental well-being, and two protective resilience factors, personal competence, and family cohesion) contributed more than 8% to the definition of the gradient of mental health status. Only one variable related to mental disorders, depression, behaved similarly. The variables of violence and problematic behavior contributed very little (less than 3%) to the definition of the two MCA dimensions.

**Table 2:** Contribution of the variables to the first two dimensions of the MCA

Variable	Dimension 1 (%)	Dimension 2 (%)
Positive mental health factors		
Mental well-being	8.9	10.8
Health-related quality of life	10.4	12.7
Resilience: personal competence	9.1	11.9
Resilience: social competence	5.4	7.3
Resilience: structured style	6.7	7.4
Resilience: social resources	7.8	8.4
Resilience: family cohesion	8.1	10.4
School resilience	6.4	6.1
Mental disorder-related factors		
Perceived stress	7.3	7.1
Depression scale	8.1	6.9
Anxiety scale	6.2	4.2
Physical fight	1.7	0.5
Bullied (victim)	1.6	0.4
Cyber-bullied (victim)	2.3	1.4
Bullying (perpetrator)	2.5	0.3
Cyber-bullying (perpetrator)	1.6	0.4
Smoking tobacco	1.8	1.3
Drinking alcohol	1.1	0.4
Getting drunk	1.3	0.8
Using cannabis	1.3	1.0

Data are represented by percentages (%). The expected average contribution is 5% (100% divided by 20 variables)

The dimension 1 explains the gradient of the mental health status of the participants, and the dimension 2 identifies the extreme values of the mental health outcomes

The cluster analysis identified three main clusters using the k-means method, with average Jaccard similarities higher than 0.90 for all of them (0.92, 0.99, 0.99); therefore, the clusters were highly stable. The three clusters are represented in the map employing the MCA (Fig. 1b) and characterized in Table 3. There were statistically significant inter-group differences in some socio-demographic characteristics, gender (*p* < 0.001), number of cohabiting children (*p* = 0.001), and birth position (*p* = 0.003), and between all mental health variables (*p* < 0.001).

**Table 3:** Description and differences between the three clusters

	Total	Cluster 1 "Good" mental health	Cluster 2 "Intermediate" mental health	Cluster 3 "Poor" mental health	*p* value
Sample size, *n* (%)	3767	993 (26%)	2553 (68%)	221 (6%)	
Socio-demographic characteristics, *n* (%)					
Gender					< 0.001
Female	1907 (51%)	478 (48%)	1316 (52%)	113 (52%)	
Male	1820 (49%)	511 (52%)	1213 (48%)	96 (44%)	
Non-binary	19 (0.5%)	1 (0.1%)	9 (0.4%)	9 (4.1%)	
Living with other children					0.001
No	951 (25%)	214 (22%)	670 (26%)	67 (30%)	
With 1 child	1852 (49%)	531 (54%)	1231 (49%)	90 (41%)	
With 2 or more children	936 (25%)	239 (24%)	633 (25%)	64 (29%)	
Position born (oldest)	1603 (44%)	460 (47%)	1068 (43%)	75 (35%)	0.003
Born in the country of residence	3443 (92%)	928 (93%)	2318 (92%)	197 (90%)	0.086
Positive mental health outcomes, mean (SD)					
Mental well-being	50.9 (8.1)	58.5 (5.2)	49 (6.1)	38.1 (9)	< 0.001
Health-related quality of life	69.4 (15.6)	84.8 (8.3)	65.5 (11.8)	42.5 (16)	< 0.001
Resilience	3.8 (0.6)	4.4 (0.3)	3.7 (0.4)	2.7 (0.7)	< 0.001
School resilience	3.7 (0.8)	4.2 (0.6)	3.5 (0.6)	2.6 (0.8)	< 0.001
Mental disorder-related outcomes, mean (SD)					
Perceived stress	5.7 (2.9)	3.2 (2.3)	6.3 (2.3)	9.7 (3)	< 0.001
Depression symptoms	6.5 (4.7)	3.1 (2.7)	7.1 (4)	14.8 (6.3)	< 0.001
Moderate-to-severe, *n* (%)	775 (22%)	28 (3%)	583 (25%)	164 (78%)	< 0.001
Anxiety disorder	5.5 (4.4)	2.7 (2.8)	6 (4)	11.8 (5.7)	< 0.001
Moderate-to-severe, *n* (%)	623 (17%)	31 (3%)	449 (18%)	143 (66%)	< 0.001

Categorical data presented as frequencies and percentages (%) and continuous data as means and standard deviation (SD); *p* value for difference between clusters. Percentages of missing values for all variables ranged from 1.2 to 7.6%. Scales: mental well-being (WEMWBS) [16], range 14–70; health-related quality of life (KIDSCREEN-10) [18], range 0–100; resilience (READ) [19], range 1–5; school resilience [20], range 1–5; perceived stress (PSS-4) [21], range 0–16; depression (PHQ-9) [23], cut-off point between moderate and severe, ≥ 10; Anxiety disorder (GAD) [26], cut-off point between moderate and severe, ≥ 10

Cluster 1 was composed of 993 (26%) adolescents. They represented the group with better mental health outcomes than the remaining clusters, i.e., the "good mental health profile." The participants in this group reported the highest mean scores in mental well-being (58.5; SD = 5.2) and HRQoL (84.8; SD = 8.3), above the normative values for these scales (51 and 71.9, respectively). They also reported the highest scores in individual and school resilience. For the mental disorder-related factors, they displayed a low level of perceived stress (3.2, SD = 2.3); the value lower than the norm of 5.4. Among the adolescents in this group, 3% presented moderate-to-severe depression and anxiety symptoms (see Table 3). In this profile, there were more males, more adolescents living with one more child, and more oldest siblings (51.6%, 54%, and 47.3%, respectively) than in the other clusters (statistically significant differences).

Cluster 2 was the largest group, containing 2553 (68%) adolescents. In this group, the mental health levels were close to the mean of the total sample, representing the "intermediate mental health profile." The adolescents in this cluster had lower scores for mental well-being, HRQoL, and resilience than those in Cluster 1 but higher than in Cluster 3. The scores for mental disorder-related factors such as stress, depression, and anxiety symptoms were higher than in Cluster 1 but lower than in Cluster 3.

Cluster 3 contained adolescents with the "poor mental health profile," with 221 subjects (6%). This group reported the worst mental health outcomes. The adolescents within this profile self-reported scores more than 10 points below the norm value in mental well-being and 29.4 points below the normal HRQoL score. They also obtained the lowest scores in the individual and school resilience scales, 2.7 (SD = 0.7) and 2.6 (SD = 0.8), respectively. Within this cluster, the perceived stress level was almost 1 point above the norm; 78% of the subjects reported moderate-to-severe depression symptoms and 66%, anxiety symptoms. This group had a lower proportion of males (44%) and a larger number of non-binary participants (4.1%) than other clusters. The profile was more common among the only children and those cohabiting with 2 or more children than in other family groups (see Table 3). It also contained the largest percentage of non-native adolescents, even though no statistically significant differences between their participation in different clusters were found (*p* = 0.086).

**Fig. 2:**
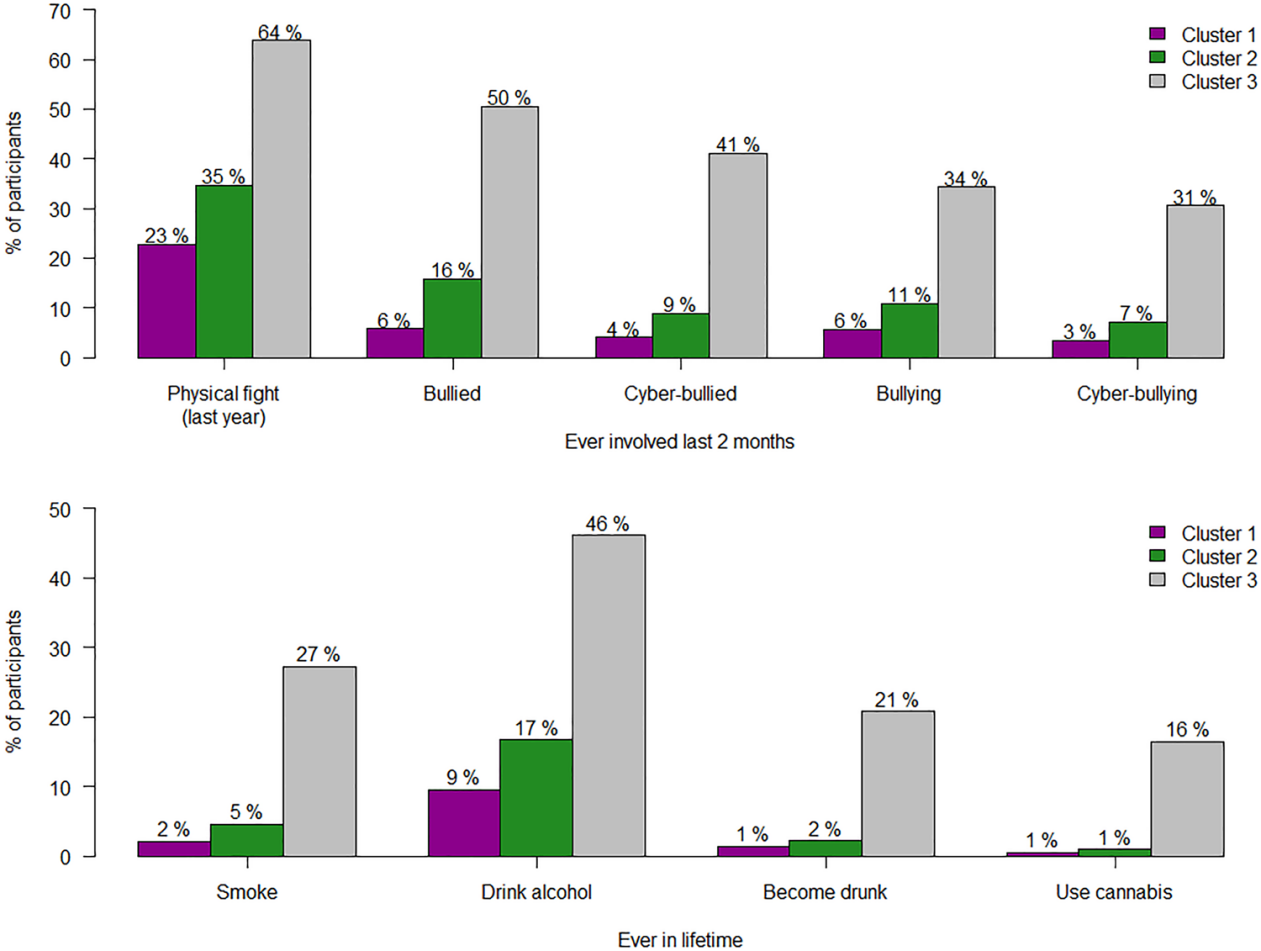
Conduct problems (fighting, bullying, cyber-bullying, and substance use) in the three clusters. Data represent the percentage of participants for each variable. All differences between clusters are statistically significant, *p* < 0.050. Cluster 1: adolescents with "good mental health"; Cluster 2: adolescents with "intermediate mental health"; and Cluster 3: adolescents with "poor mental health"

The same trend was observed for conduct problems and substance use. The group of adolescents with the "good mental health profile" contained the lowest percentage of subjects with these problems, followed by the participants with the "intermediate mental health profile." The group with the "poor mental health profile" showed the largest proportion of adolescents with conduct problems and substance use behavior. All differences were statistically significant (*p* < 0.050) (Fig. 2).

## Discussion

This study provides an overview of the state of mental health of adolescents across Europe, following a comprehensive assessment of both positive mental health and mental disorder-related outcomes. In general, the participants showed positive mental health levels within the normal value range. However, 22% and 17% of them self-reported moderate-to-severe levels of depression and anxiety symptoms, respectively. Balazs et al. [31] have reported the rates of depression ranging from 7.1 to 19.4% in European adolescents, while Merikangas et al. [32] have obtained the generalized anxiety disorders rates of 2.2% for the USA youths. The rates of depression and anxiety estimated in our study may be misaligned, because they were based on self-reports; the results were not corroborated by personal assessment by a clinical expert. However, our data indicate that approximately 2 in 10 European adolescents self-report depression or anxiety at relatively high levels. After rounding the percentages, the results show that 1 in 10 adolescents’ reports being a victim of bullying or cyber-bullying; 1 in 14 admits to being a perpetrator of bullying, and 1 in 10 engages in cyber-bullying. Sixteen percent of the subjects self-report having consumed alcohol, and less than 5% consuming other substances (smoking tobacco, cannabis). To reduce these numbers, it is advisable to promote mental health interventions and prevent mental disorders in adolescents.

Our exploration of adolescent mental health resulted in three distinct mental health profiles. In the most prevalent ("intermediate mental health") profile, the scores for the positive and negative mental health variables were similar to those for the total sample of adolescents. This profile can be compared with the "symptomatic but content" profile of the dual-factor model [33]. In both groups, the adolescents show intermediate levels of subjective well-being, but the groups display different levels of psychopathology. The “symptomatic but content” group shows a high psychopathology level [33], which might exceed the cut-off points for mental health problems. The adolescents in the “intermediate mental health” profile show mild-to-moderate levels of psychological distress that, in most cases, do not exceed the threshold in psychopathology screening. In other words, this group is not, in general, distinguished by its high levels of well-being or high levels of psychological distress. Rather, it is a group with a near-average level of mental well-being and mild-to-moderate depression, anxiety, and stress symptoms. However, they often do not receive specific support, because their levels of well-being, quality of life, and resilience are similar to the average [2].

The second most frequent mental health profile (26% of the participants), "good mental health," groups the adolescents whose levels of positive mental health are higher and their mental disorder-related scores are lower than average. Antaramian et al. [13] have observed that the positive mental health group in their study demonstrated higher school participation levels and better grades than the others. Similarly, the "good mental health" profile in our study showed the highest level of school resilience. In terms of socio-demographic characteristics, it is in this profile that we found the highest percentage of male adolescents (first-born and native), compared to the other two profiles. Our results for gender and birth order are in agreement with those reported by Zilanawala, Sacker and Kelly [34]. Their study has found that English male adolescents report better indicators of positive mental health (i.e., quality of life and mental well-being) than their female peers. Moreover, they have shown that first-born individuals enjoy better mental health than their second or subsequent siblings, which is consistent with our data.

Finally, the third profile, the "poor mental health," constituted 6% of the examined sample. This profile included adolescents that reported lower than average levels of positive mental health and high levels of mental disorder-related factors. Moreover, this profile revealed the highest frequency of behavioral problems. In agreement with our results, the studies by Moore et al. [35] and Kim et al. [12, 36] have shown that the frequency of conduct problems was higher in the health profiles with more mental disorder-related factors. Similarly, Keyes [35, 36] has found a higher prevalence of such problems (missing school, getting arrested, smoking cigarettes, and smoking marijuana) in the "mentally unhealthy" (the term used in that study) adolescent group, constituting also 6% of their sample.

Other studies have also reported results similar to those presented here. However, they have employed different empirical approaches to identify the mental health profiles (e.g., latent profile analysis, LPA) and different, often smaller, sample sizes [37]. The methods used in the definition of mental health profiles for adolescents are of great importance. The synergy between reduction technique (MCA) (explaining the relationship between positive mental health and mental disorder-related outcomes) and classification method (cluster analysis) is invaluable in defining these profiles. These techniques do not require predefined assumptions; moreover, thanks to the large sample size used here, the derived clusters are highly stable. The main difference between our study and the studies of Moore et al. [35] and Reinhardt et al. [37] is the number of health profiles; we found three such profiles, while they have reported four. The "languishing" profile (low well-being and low distress levels) described by Reinhardt et al. [37] did not emerge in our sample. Moore [35] has argued that the absence of the languishing profile might be explained by its low frequency among the adolescents, making its detection, using the current analytical methods, difficult.

Typically, the profile associated with the largest number of adolescents is the "complete mental health" group [33]. In contrast, in our study, the largest group was the "intermediate mental health" profile. The cause of this discrepancy is uncertain, and further research is needed to confirm this finding. Possible reasons for these differences might include the characteristics of our sample, where the mean age of participants is 12, and the adolescents come from Europe. The previous studies [33, 38] have explored the mental health of adolescents older than 12 or living outside Europe. Some studies have used different analytical techniques to generate the profiles, such as cut-off classifications or latent profile analyses, which could lead to different results. Furthermore, more cases of mental health problems have been reported in the adolescent population over the years [39, 40]; this could either reflect increasing morbidity or a greater willingness on the part of adolescents to communicate their emotional distress. Given their positive attitudes, many adolescents within the “intermediate mental health” profile may not receive the needed therapeutic support; they function well in their daily lives. Thus, the relevance of their symptoms may be overlooked. To avoid this, universal mental health promotion and preventative interventions should include the entire adolescent population and not just the most vulnerable groups or those already diagnosed with mental disorders. As Huppert [6] argues, the "evidence from epidemiology suggests that if we only use this targeted approach, there will always be many new cases of disorder, as most of those who develop the disorder are from the general population" [intermediate group in our case].

Universal interventions targeting the general public effectively promote positive mental health outcomes [41], regardless of the vulnerability of the population to mental disorders. Besides, targeted interventions directed at the groups whose vulnerability is higher than average can be conducted in a complementary manner. In the future, the best mental health profile should include the largest number of adolescents, since it is associated with the most positive results (see [42] for a review).

A key aspect of this study is demonstrating, using statistical classification tools, the hegemonic weight of positive mental health dimensions in the profile identification for European adolescent populations. Traditionally, the characterization of the mental health of adolescents has been colored by a negative perception created by assessing the symptoms of depression, anxiety, or stress. However, the statistical models used here show that the ability of the adolescents to manage the interactions within their social environment carries considerable weight in the construction of their mental health profiles. Their personal resources measured using positive scales such as well-being, resilience, and quality of life, emerged as the key determinants of a healthy process of growing up. In the same vein, Kim et al. [12] have used a series of ANOVAs to conclude that, in assessing satisfaction with life among Korean adolescents, the perception of personal strengths was more important than their perception of mental distress. An earlier study by Kim et al. [11] had found that positive mental health status is a better predictor of subjective well-being than behavioral and emotional symptoms. Thus, the interventions designed to promote subjective well-being should be considered complementary and as worthy of implementation as interventions whose object is to prevent mental disorders [13].

Schools that implement mental health promotion programs are aligned with the idea that focusing on the positive aspects of mental health increases mental well-being and prevents mental distress. Internal strengths, such as resilience, and collective assets, such as school resilience, can act as protective factors mitigating the negative impact of stressors. They can form a buffer against the development of psychological disorders. The UPRIGHT program is an example of a school-based intervention to increase resilience and thus improve the mental health of adolescents. Indirectly, it can also reduce the long-term symptoms of mental disorders [43]. A recent study of positive interventions in schools [44] has shown that these interventions are universally beneficial. They are particularly helpful among the adolescents described by their teachers as most in need of mental health care. Our results show that the "intermediate mental health" and "poor mental health" groups could benefit from such school-based interventions. Applying an intervention to the entire student body (i.e., universal interventions) reduces the potential for stigmatization based on mental health issues. These interventions also reduce the sense of loneliness of students with mental health problems. Such students often feel alienated and stigmatized, prefer not to ask for help, and remain silent, not to be labeled as problematic [44]. In agreement with the previous reports [35], our study shows the importance of including positive mental health scales in school-based mental health assessment programs. The introduction of such scales helps avoid the bias created by focusing on the negative (i.e., deficit-oriented) approach [11, 12].

This study did not use some of the socio-economic, family, cognitive, and physical health variables in the analyzed data pool. Including these data would have improved our understanding of the mental health profiles and helped in explaining the differences. Platt et al. [44] have reported that teachers consider economic deprivation a major cause of poor mental health in adolescents. A study by Zilanawala et al. [34] has shown that 6% of UK adolescents who matched their "poor health profile" had low cognitive skills and were more likely to acquire alcohol and tobacco use habits. They also displayed increased levels of antisocial behavior and low self-esteem.

In the current study, the data were collected using self-administered questionnaires, which weakens their reliability due to a bias toward monomethod and social convenience. However, it is recognized that adolescents are the ideal source of information on their own mental state [45]. The cross-sectional design of our study does not let us assess the structural stability of the obtained profiles or to infer causal relationships between the variables. According to the previous studies [38, 46] the adolescents move to another mental health profile within a few years (after 1 year and 4 years of follow-up, respectively). Therefore, it is expected that most of the adolescents in our sample will not remain in the same mental health profile during the next few years, with the girls worsening their average mental health [47]. Although the sampling limitations do not allow a full generalization of the results, the large and diverse group studied here supplies valuable insights from diverse, multicultural European contexts. Our sample includes 51% of females and 92.1% of adolescents born in the country of residence, which is similar to the data found in the Eurostat database (48.8% and 93.8%, respectively) [48].

The results of this study open up some new research prospects. Future studies could explore the responsiveness to various mental health promotion interventions, depending on the mental health profile. Furthering the knowledge of the mental health status of European adolescents can help the policymakers decide on the implementation of specific types of mental health promotion interventions. Furthermore, this baseline information could also help understand the effect of the COVID-19 pandemic on the future mental health of adolescents. The adolescents associated with profiles with the highest levels of well-being and lowest vulnerability to mental disorders could be those who will navigate the pandemic journey with the least negative consequences. The results of this study can also serve as the basis for testing the effectiveness of preventive interventions, such as the UPRIGHT program, in the general population of early adolescents.

## Conclusion

Working within the UPRIGHT research project framework, the present study identified three different mental health profiles in a large sample of adolescents from five European countries. It explored both the positive mental health aspects and mental disorder-related factors. The most prevalent profile was associated with intermediate mental health; this group showed standard levels of positive mental health indicators and mild-to-moderate mental disorder symptoms. The information gained in this study supports the need to implement school-based and universal programs to promote mental well-being and prevent mental disorders in adolescents.

## Supplementary Information

Below is the link to the electronic supplementary material.

Supplementary file1 (PDF 190 KB)

## Data Availability

Not applicable. The UPRIGHT consortium has the commitment with the European Commission to share study datasets (except that identifying/confidential patient data) in publicly available repositories. We are still working in the way we are going to make these data available (type of data and platform). The project is ongoing and datasets have not yet been completed, which is expected for the end of year 2021.
