# Supplementary material for: Relevance of well-being, resilience, and health-related quality of life to mental health profiles of European adolescents: results from a cross-sectional analysis of the school-based multinational UPRIGHT project
Source: Soc Psychiatry Psychiatr Epidemiol. 2021 Aug 21;57(2):279–91. doi: 10.1007/s00127-021-02156-z (PMC8379563; doi:10.1007/s00127-021-02156-z)
Supplement: Supplementary file 1 — Supplementary file1 (PDF 190 KB) [file 127_2021_2156_MOESM1_ESM.pdf]

Article title

"Relevance of well-being, resilience and health-related quality of life to mental health profiles of European adolescents: results from a cross-sectional analysis of the school-based multinational UPRIGHT project"

Journal name

Social Psychiatry and Psychiatric Epidemiology

Author names

Carlota Las Hayas*, Maider Mateo-Abad, Itziar Vergara, Irantzu Izco-Basurko, Ana González-Pinto, Silvia Gabrielli, Iwona Mazur, Odin Hjemdal, Dora Gudrun Gudmundsdottir, Hans Henrik Knoop, Anna S. Olafsdottir, Ane Fullaondo, Nerea González, Javier Mar Medina, Dominik Krzyżanowski, Roxanna Morote, Frederick Anyan, Mette Marie Ledertoug, Louise Tidmand, Unnur B. Arnfjord, Ingibjorg Kaldalons, Bryndis Jona Jonsdottir, Esteban de Manuel Keenoy on behalf of the UPRIGHT Consortium.

*Corresponding author

Affiliations

Carlota Las Hayas, PhD* clashayas@kronikgune.org	• Kronikgune Institute for Health Services Research, Ronda de Azkue 1 torre del Bilbao Exhibition Centre, Barakaldo 48902, Spain
Maider Mateo-Abad, PhD MAIDER.MATEOABAD@osakidetza.eus	• Kronikgune Institute for Health Services Research, Basque Country, Spain;<br>• REDISSEC (Health Services Research on Chronic Patients Network), Basque Country, Spain
Itziar Vergara, PhD MD MARIAICIAR.VERGARAMITXELTORENA@osakidetza.eus	• Kronikgune Institute for Health Services Research, Basque Country, Spain;<br>• Biodonostia Health Research Institute, San Sebastián, Spain;<br>• REDISSEC (Health Services Research on Chronic Patients Network), Basque Country, Spain
Irantzu Izco-Basurko, MS iizco@kronikgune.org	• Kronikgune Institute for Health Services Research, Basque Country, Spain
Ana González-Pinto, Prof. PhD MD ANAMARIA.GONZALEZ-PINTOARRILLAGA@osakidetza.eus	• Osakidetza Basque Health Service, Araba University Hospital, Basque Country, Spain;<br>• UPV/EHU<br>• CIBERSAM (CIBER of Mental Health Area);<br>• Bioaraba Health Research Institute, Vitoria-Gasteiz, Spain
Silvia Gabrielli, PhD sgabrielli@fbk.eu	• Bruno Kessler Foundation. Via Santa Croce 77, Trento 38122, Italy
Iwona Mazur, PhD mazuri@interia.pl	• Daily Centre for Psychiatry and Speech Disorders (Poland).<br>• Wroclaw Medical University (Poland).
Odin Hjemdal, Prof. PhD odin.hjemdal@ntnu.no	• Norwegian University of Science and Technology, Hogskoleringen 1, Trondheim 7491, Norway

Dora Gudrun Gudmundsdottir<br>dora@landlaeknir.is	• Directorate of Health, Baronsstig 47, Reykjavik 101, Iceland
Hans Henrik Knoop, Prof. PhD<br>knoop@edu.au.dk	• Aarhus University, Nordre Ringgade 1, Aarhus C 8000, Denmark<br>• Optentia Research Focus Area, P O Box 1174, North-West University, Vanderbijlpark, 1900 , South Africa
Anna S. Olafsdottir, Prof. PhD<br>annaso@hi.is	• University of Iceland, School of Education, Saemundargotu 2, Reykjavik 101, Iceland
Ane Fullaondo, PhD<br>afullaondo@kronikgune.org	• Kronikgune Institute for Health Services Research, Ronda de Azkue 1 torre del Bilbao Exhibition Centre, Barakaldo 48902, Spain
Nerea González, PhD<br>ngonzalez@kronikgune.org	• Kronikgune Institute for Health Services Research, Ronda de Azkue 1 torre del Bilbao Exhibition Centre, Barakaldo 48902, Spain<br>• Osakidetza Basque Health Service, Barrualde-Galdakao Integrated Health Organisation;<br>• REDISSEC (Health Services Research on Chronic Patients Network), Basque Country, Spain
Javier Mar, PhD MD<br>FRANCISCOJAVIER.MARMEDINA@osakidetza.eus	• Osakidetza Basque Health Service, Debagoiena Integrated Health Organisation, Research Unit, Arrasate-Mondragón, Spain;<br>• Biodonostia Health Research Institute, San Sebastián, Spain;<br>• REDISSEC (Health Services Research on Chronic Patients Network), Basque Country, Spain;<br>• Kronikgune Institute for Health Services Research, Ronda de Azkue 1 torre del Bilbao Exhibition Centre, Barakaldo 48902, Spain
Dominik Krzyżanowski<br>dominik.krzyzanowski@dolnyslask.pl	• Division of Medical Social Science Wroclaw Medical University, Poland<br>• Department of Health, Lower Silesia Voivodeship Marshal Office, Poland
Roxanna Morote, PhD<br>roxanna.morote@ntnu.no	• Norwegian University of Science and Technology, Hogskoleringen 1, Trondheim 7491, Norway<br>• Catholic University of Peru, Avenida Universitaria s/n, 18, Lima, Peru
Frederick Anyan, PhD<br>frederick.anyan@ntnu.no	• Norwegian University of Science and Technology, Hogskoleringen 1, Trondheim 7491, Norway
Mette Marie Ledertoug, PhD<br>mele@edu.au.dk	• Aarhus University, Nordre Ringgade 1, Aarhus C 8000, Denmark
Louise Tidmand<br>Loti@edu.au.dk	• Aarhus University, Nordre Ringgade 1, Aarhus C 8000, Denmark
Unnur B. Arnfjord<br>ubj@hi.is	• University of Iceland, School of Education, Saemundargotu 2, Reykjavik 101, Iceland
Ingibjorg Kaldalons, PhD<br>ingakald@hi.is	• University of Iceland, School of Education, Saemundargotu 2, Reykjavik 101, Iceland
Bryndis Jona Jonsdottir<br>bryndisjona@hi.is	• University of Iceland, School of Education, Reykjavík (Iceland)

Esteban de Manuel Keenoy, MD<br>edemanuel@kronikgune.org	• Kronikgune Institute for Health Services Research, Ronda de Azkue 1 torre del Bilbao Exhibition Centre, Barakaldo 48902, Spain
**On behalf of the UPRIGHT consortium**	Iñaki Zorrilla-Martínez (INAKI.ZORRILLAMARTINEZ@osakidetza.eus)<br>• Bioaraba Health Research Institute, Neurosciences Research Area, Vitoria-Gasteiz, Spain<br>• Osakidetza Basque Health Service, Araba University Hospital, Vitoria-Gasteiz, Spain<br>• University of the Basque Country UPV-EHU, Vitoria-Gasteiz, Spain;<br>• CIBERSAM (CIBER of Mental Health Area);<br>• Institute of Health Carlos III, Madrid, Spain<br><br>Patricia Pérez-Martínez-de-Arrieta (PATRICIA.PEREZMARTINEZDEARRIETA@osakidetza.eus)<br>• Osakidetza Basque Health Service, Araba University Hospital, Basque Country, Spain<br><br>Igor Larrañaga (Igor.LarranagaUribetxebarria@osakidetza.eus)<br>• Kronikgune Institute for Health Services Research, Ronda de Azkue 1 torre del Bilbao Exhibition Centre, Barakaldo 48902, Spain<br>• Osakidetza Basque Health Service, Debagoiena Integrated Health Organisation, Research Unit, Arrasate-Mondragón, Spain<br><br>Sara Carbone (scarbone@fbk.eu)<br>• Bruno Kessler Foundation. Via Santa Croce 77, Trento 38122, Italy<br><br>Silvia Rizzi (srizzi@fbk.eu)<br>• Bruno Kessler Foundation. Via Santa Croce 77, Trento 38122, Italy<br><br>Valeria Donisi (vdonisi@fbk.eu)<br>• Bruno Kessler Foundation. Via Santa Croce 77, Trento 38122, Italy<br>• University of Verona. P.le L.A. Scuro, 10, Verona 37134, Italy<br>Hrefna Pálsdóttir (hrefnapals@hi.is)<br>• University of Iceland, School of Education, Saemundargotu 2, Reykjavik 101, Iceland<br>Alda Ingibergsdóttir (aldai@hi.is)<br>• University of Iceland, School of Education, Saemundargotu 2, Reykjavik 101, Iceland

E-mail address of the corresponding author

clashayas@kronikgune.org

Pearson correlation coefficients for the continuous variables for males and females are presented in Table 4.

Table 4

Pearson correlation between positive mental health factors and mental disorder-related factors

	Well-being	HRQoL	Resilience	School resilience	Stress	Depression	Anxiety
Well-being	1.00	0.68	0.70	0.48	-0.50	-0.44	-0.36
HRQoL	0.72	1.00	0.71	0.50	-0.61	-0.59	-0.51
Resilience	0.72	0.75	1.00	0.53	-0.51	-0.46	-0.38
School resilience	0.50	0.57	0.60	1.00	-0.40	-0.35	-0.30
Stress	-0.59	-0.69	-0.58	-0.42	1.00	0.53	0.51
Depression	-0.56	-0.70	-0.56	-0.43	0.62	1.00	0.71
Anxiety	-0.48	-0.60	-0.44	-0.33	0.60	0.72	1.00

White area represents correlations for females and grey area, for males. Scales: mental well-being (WEMWBS) [1], HRQoL, health-related quality of life (KIDSCREEN) [2], resilience (READ) [3], school resilience [4], perceived stress (PSS-4) [5]; depression (PHQ-9) [6], and anxiety disorder (GAD) [7].

All correlations were statistically significant. The correlations for female participants were slightly higher than for the males, especially the negative correlation of depression or anxiety scores with positive mental health outcomes. The highest positive correlation coefficients were found for well-being, HRQoL, and resilience (*r* coefficients from 0.68 to 0.75), and for anxiety and depression (r = 0.72 and r = 0.71, for females and males, respectively). The highest negative correlation was between the HRQoL and perceived stress (r = -0.69) and the depression scale (r = -0.70), for female adolescents only.

Conclusion

The correlations between positive aspects of mental health and mental disorder-related factors were negative and of moderate strength. These results suggest that such associations, although generally inversely related, are highly variable. The analysis of these correlations indicates that the positive mental health variables are not the opposites of those linked to mental disorders but are different and related concepts. Thus, the interventions designed to promote subjective well-being should be considered complementary and as worthy of implementation as interventions whose object is to prevent mental disorders.

Reference list

1.Tennant R, Hiller L, Fishwick R et al (2007) The Warwick-Edinburgh mental well-being scale (WEMWBS): development and UK validation. Health and Quality of life Outcomes 5(1):63.

2. The KIDSCREEN Group Europe (2006) The KIDSCREEN Questionnaires. Quality of life questionnaires for children and adolescents. Lengerich: Pabst Science Publishers.

3. Hjemdal O, Friborg O, Stiles TC et al (2006) Resilience predicting psychiatric symptoms: A prospective study of protective factors and their role in adjustment to stressful life events. Clinical Psychology & Psychotherapy: An International Journal of Theory & Practice 13(3):194-201.

4. Morote R, Anyan F, Las Hayas C et al (2020) Development and validation of the theory-driven School Resilience Scale for Adults: Preliminary results. Children and Youth Services Review 119:105589.

5. Cohen S, Kamarck T, Mermelstein R (1983) A global measure of perceived stress. J Health Soc Behav 24(4):385-396.

6. Kroenke K, Spitzer RL, Williams JB (2001) The PHQ-9: validity of a brief depression severity measure. J Gen Intern Med 16(9):606-613. doi:10.1046/j.1525-1497.2001.016009606.x

7. Löwe B, Decker O, Müller S et al (2008) Validation and standardization of the Generalized Anxiety Disorder Screener (GAD-7) in the general population. Med Care:266-274.
